# Fishy culture in a changing world

**DOI:** 10.1098/rstb.2024.0130

**Published:** 2025-05-01

**Authors:** Culum Brown, Michael Webster

**Affiliations:** ^1^ School of Natural Sciences, Macquarie University, Sydney, New South Wales 2109, Australia; ^2^ University of St Andrews, St Andrews KY16 9AJ, UK

**Keywords:** migration, traditions, social information, social learning, social transmission

## Abstract

Animal cultures have been recognized by researchers since the mid-twentieth century, with research interest growing over the past few decades. Recently, we have realized that animal cultures have important ecological consequences, shaping mating preferences, adaptation to urban environments and the persistence of migration routes. The study of culture therefore transcends traditional, curiosity-driven research; it has real-world conservation relevance. This review centres on culture in fishes and its intersection with conservation. Culture depends upon social learning, and a large literature establishes that fishes exhibit social learning in a diverse range of contexts. Moreover, laboratory experiments demonstrate a capacity for culture in fishes, while studies of wild populations provide evidence of natural cultures, specifically the maintenance of traditional migration routes in some reef fishes. Here, we argue that such cultures are likely more widespread but overlooked and should be the target of wider research interest and conservation effort. We also argue that there is greater scope to leverage findings from laboratory studies of fish cultural transmission to better equip reintroduced fish stocks with behaviours that might enhance post-release survival. Fish cultures warrant investigation in their own right and have the potential to inform how we understand and conserve animal cultures more widely.

This article is part of the theme issue ‘Animal culture: conservation in a changing world’.

## 1. Introduction

Animal cultures have been recognized by behavioural biologists since the mid−twentieth century, with research interest growing markedly over the last two decades. A widely accepted definition of culture, one that we adopt here, recognizes that it comprises behaviours that are shared by members of a community that are spread and maintained by socially learned and socially transmitted information [1]. Much early work on animal culture focused upon primates. More recently, however, we have come to realize that non-primate species have a capacity for culture too and that this has important ecological consequences, shaping for example, mating preferences [2], adaptation to urban environments [3] and the persistence of migration routes [4].

This review centres on culture in fishes. Culture depends upon social learning, and we first discuss the large literature establishing that fishes exhibit social learning in a diverse range of contexts [5]. Laboratory experiments demonstrate a capacity for culture in fishes, and there is evidence of cultural transmission under natural conditions in fishes too, with the most compelling concerning the maintenance of traditional migration routes in some reef-dwelling fishes. We argue that such route-establishment cultures are likely more widespread in fishes but have been overlooked and should be the target of wider research interest and conservation effort. Many fish species are imperilled largely owing to anthropogenic impacts such as climate change, river regulation and over-fishing. The proportion of fish stocks that are over-fished continues to increase, with 37.7% of global fish stocks considered unsustainably fished in 2021, up from 10% in 1974 [6], and 25% of assessed freshwater fishes are threatened with extinction [7]. Thus, there is urgent need for innovative management solutions. Here, we also consider applications to fish stock reintroductions, a widely used conservation tactic that is associated with high mortality of stocked fish. We suggest that recognizing social learning and culture may not only help identify important biodiversity and aid fisheries management but also offer avenues for conservation solutions [8,9].

## 2. Learning and social learning in fishes

Fish are often viewed as mindless automatons but if fact research over several decades has shown that their capacity for learning and memory is no different from terrestrial vertebrates [10]. This should come as no surprise given that learning provides the necessary behavioural flexibility to match prevailing ecological conditions. Fish can learn about what and where to eat, migration routes, the identity of predators and even whom to mate with [11–13].

Fish can not only learn about their environment directly by trial and error but they also rely on information emanating from their social environment. Social learning occurs when an animal learns by watching or interacting with another animal and/or its products [14]. These two sources of information are often referred to as private and public information, respectively, and fishes often optimize the balance of these two sources of information to reach fitness optima [15]. This makes sense because gathering information about the environment on one’s own is extremely time-consuming and potentially dangerous. Such wasted time could be better spent on other activities and any unnecessary activity increases the chance of being exposed to predation. On the positive side, an individual that gathers information directly from the environment can be assured that it is accurate. Relying on social learning, on the other hand, can be rapid and efficient, but there is a chance that the information may be out of date or obsolete. When the information is out of date, this can cause reliance on social learning to become maladaptive [16,17]. With these costs and benefits in mind, it becomes apparent that a mix of these strategies is likely to be optimal, depending on the context [18–20]. If the environment varies temporally, for example, then the balance would be tipped in favour of individual learning, whereas moderate-to-low environmental variation would favour social learning [21]. The optimal choice might also depend on the social context and prior experience [22]. Moreover, variation in the risk-taking behaviour of individuals or species can also shift the balance of these two sources of information. Nine-spined sticklebacks, *Pungitius pungitius*, for example, tend to rely more heavily on social learning rather than private information than do three-spined stickleback, *Gasterosteus aculeatus*, perhaps because they are not as heavily armoured and thus more prone to predation [23,24]. Similarly, variation in boldness within a species can influence how individuals trade-off social and private information [25]. Recent experiments have also examined this trade-off between public and private information in field experiments using fathead minnows, *Pimephales promelas*, in an anti-predator context [26].

Social learning appears to be ubiquitous across all behavioural domains in fish (see reviews by [27,28]). Experiments conducted on rainbowfish, *Melanotaenia* spp., learning to avoid a model trawl apparatus, for example, found that individuals seemed to rely on information provided by their shoalmates as to the whereabouts of the escape route in the net (local enhancement) or were motivated by witnessing the successful escape of a fellow shoal member (stimulus enhancement; [29]). A follow-up experiment found that shoals comprised of more individuals also learned more quickly to avoid the trawl by using the escape route than smaller groups [30]. Further studies using zebrafish, *Danio rerio*, and guppies, *Poecilia reticulata*, seeded groups with pre-trained individuals (demonstrators), resulting in the rapid escape of the entire group relative to those groups that were not seeded [31,32]. Fish can also learn to recognize and avoid real predators via social learning, relying on both visual and chemical cues. When one fish displays behavioural signs of alarm, this behaviour can rapidly spread through the rest of the shoal (social contagion; [33]). Similar responses occur when fish are exposed to alarm cues emanating from injured conspecifics, which can then be paired with the odour of a specific predator [34]. Fish can also learn the location of foraging patches via social learning. In the classical paradigm, demonstrator fish are pretrained to use one of two doors to access food hidden behind a partition. These demonstrators are then transplanted into small groups of naive fish. Control schools contain sham demonstrators. The schools are then tested to see how quickly they locate the foraging patch and which door they use to access it. Universally, shoals containing demonstrators learn the task faster than control groups (e.g. [35]). Finally, fish can learn to copy mate choice preferences by watching others choose before them [36].

The pathway through which social information is likely to be transmitted can be predicted by examining social relationships. By quantifying social interactions within groups, one can generate a social network wherein the nodes represent individuals and the weighted lines connecting them (edges) represent the frequency of social contact [37]. Croft *et al*. [38] tracked social interaction in wild groups of guppies in Trinidad and found that females in particular formed stable social interactions over periods longer than 7 days. Later studies also found evidence of stable male–male dyads but genetic analysis suggested that none of the relationships were influenced by relatedness [39]. Moreover, the positions that individuals adopted within the network were consistent and predicted by their tendency to be social, as well as by sex-specific social preferences [40]. Analyses of social networks in a range of elasmobranch and teleost species have repeatedly found evidence of non-random social interactions between individuals [41].

**Figure 1.**
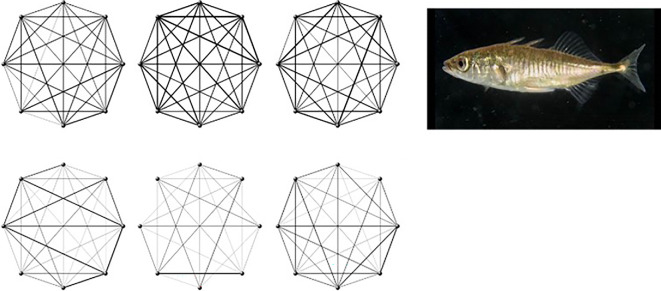
Social (association) networks for experimentally created shoals of sticklebacks. Each plot shows a replicate shoal. The points represent individual fish and the thickness of the connecting lines indicates how frequently they interacted, with thicker lines indicating more frequent interaction. The upper row of plots show shoal networks for shoals tested in open environments and the lower plots show shoals tested in structurally more complex environments. In the latter cases, the networks were more heterogeneous and they better predicted the order in which fish discovered hidden prey patches, with individuals being more likely to find these if a closely connected group mate had already done so. Based on Webster *et al*. [42].

While it is theoretically possible for social (public) information to be transferred by single chance interactions, this is far more likely to occur between individuals that interact regularly. Thus, by quantifying social networks one should be able to predict how information is likely to move through a shoal (figure 1). Webster *et al*. [42] tested this prediction by conducting a foraging experiment wherein three-spined sticklebacks had to discover the location of a hidden foraging patch. In open environments, fish formed larger groups with a more homogenous social structure. Here, social network structure was not strongly related to food patch discovery. In structurally complex environments, shoals were smaller and social networks contained cliques of more strongly connected individuals. The order in which the sticklebacks discovered the patch could be explained by the social network properties in each experimental shoal. Similar experiments that investigated the transmission of information through social networks revealed that network structure can predict the order in which individuals discover resources—in this case food patches hidden in artificial tubes that the fish had to learn how to access—even if the fish subsequently learn how to access the food asocially [43]. In other words, fish that interact frequently tend to find resources together, a social effect, but they do not necessarily work together to discover how to exploit them. This study revealed a subtle social influence that enhances learning indirectly. A follow-up study to this revealed that familiarity affected social network structure in sticklebacks, with familiar fish tending to associate with each other more frequently than unfamiliar fish. The same indirect social effect upon patch discovery was apparent, with individuals being more likely to discover new patches (whether they contained food or were empty) if a familiar shoal mate had already found them [44].

The manner in which information passes through social groups typically falls into two categories. The first category is when information passes through an age group or from peer to peer, and this is often referred to as horizonal transmission. When information moves between age groups then it is referred to as vertical transmission. Vertical transmission of information is particularly interesting because it provides a method by which information can flow from one generation to the next and can result in consistent behaviours within populations over time. These idiosyncratic population-specific behaviours are often referred to as culture.

## 3. Culture: Evidence from the lab and in the wild

**Figure 2.**
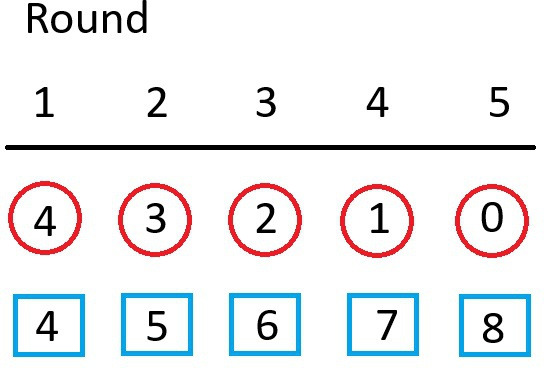
Typical transmission chain design where the given number of demonstrators in the social group (circles) are gradually removed and replaced with naive observers (squares). Here, the experiment starts with four demonstrators and four observers. By round five, none of the original pre-trained demonstrators remain in the social group.

Some of the early lab-based studies attempted to illustrate how social learning processes could lead to the development of culture. In the classic methodology, transmission chains are established wherein a preference to perform a certain behaviour is first seeded into a group by introducing pre-trained demonstrators. These demonstrators then influence the behaviour of the group and over time they are replaced by naive individuals. The fundamental idea here is that knowledgeable individuals within a population are gradually lost over time and replaced by newly recruiting individuals. This is reminiscent of elephant societies where the matriarch is in possession of key survival information, such as the location of water holes during times of drought, and her daughters learn the information by accompanying her, then taking her place as knowledgeable matriarch once she dies [45,46]. This approach works surprisingly well in fish, possibly because they are under strong selective pressure to school; thus they are highly motivated to ensure that their behaviour aligns with their school mates (conformity; [35,47]). Laland and Williams [48] adopted the transmission chain approach to study foraging routes in guppies (figure 2). Here, fish were trained to take one of two routes to locate a foraging patch. These founder demonstrators were gradually replaced over several days with naive fish. Three days after all the original founders had been fully replaced, the schools still showed strong preferences for the route learned from the founders. Interestingly, the cultural preference for taking the socially learned route was still maintained even when the alternative route was more efficient [16]. Stanley *et al*. [49] also used the transmission train methodology to examine social learning in a foraging task that is very difficult to learn via individual learning in both guppies and platies, *Xiphophorous maculatus*. The focus on a difficult task here is important because some have argued that the best evidence for social learning would stem from studies where the task is extremely difficult to learn on one’s own, but can be learned socially [50]. Similar methods have been used to examine escape responses by zebrafish in response to a model trawl apparatus [32].

The transmission chain experiments approximate the sorts of processes that we expect to see in wild fish populations, but studying these processes is extremely difficult under field conditions. Nonetheless, it is likely that fish have cultural traditions that are passed on in this way, particularly in the context of movement and migration [51]. Many fish, for example, have traditional breeding, feeding and resting grounds, and the migration routes taken between these locations are often highly conserved over multiple generations [52]. Arguably, the best evidence comes from the daily migration routes adopted by French grunts, *Haemulon flavolineatum*, in the Virgin Islands. The daytime resting places of social groups of grunts is specific to a given coral head or group of sea urchins and, as dusk descends, they head out to forage and feed on invertebrates at adjacent seagrass beds , and return at dawn. The route they adopt to move back and forth is also highly conserved. Even though school members seldom exceed 2 years of age, the preferences for specific coral head resting spots persists for at least 3 years [53]. Helfman and Shultz [54] conducted a very clever transplant experiment wherein naive individuals were integrated into existing groups. In the control groups, fish were transplanted but all of the original residents were removed. Results showed that when transplanted fish had the opportunity to interact with residents, they adopted the local foraging path, albeit with a little bit of zig-zagging. In contrast, control fish maintained similar routes and headings that they would have used had they been at their original collection location. Copper sweeper, *Pempheris schomburgkii*, which also inhabit the same reefs, similarly show consistent twilight migrations [55], as do a number of Hawaiian reef fishes [56]. It is probable that such behaviour is common in coral reef fishes around the world.

Specific migration routes are also common to access specific breeding grounds. Brown surgeonfish, *Acanthurus nigofuscus*, have both daily migration routes associated with foraging and also have spawning migrations. Close observations suggest that not only is the route passed on socially but so too are seemingly idiosyncratic movements, such as body pitches and rolls, along the way [57]. This high-fidelity replication of behaviour strongly suggests that imitation may be at play rather than local or stimulus enhancement. Warner [58,59] also conducted transplant experiments while studying the breeding migrations in bluehead wrasse, *Thalassoma bifasciatum*, and found that not a single new breeding location emerged or was lost across 22 locations over 12 years of observation! Collectively, these observations strongly suggest that both the locations and the routes to access breeding grounds are passed on from generation to generation via social learning, resulting in persistent cultural traits. We suggest that further research into the role of culture in shaping route-following in other species would be worthwhile. Much of the key research on this topic took place in the late twentieth century. There is much to be learned from using modern tagging and tracking approaches [60–62] to explore fine-scale space use in species that undergo daily and seasonal migrations. Coupling such approaches with appropriately designed translocation experiments will provide insights into the distribution of migration traditions in fish species.

## 4. Social learning, culture and conservation

Animal culture has important implications for individual fitness and thus population resilience and as such it ought to be of concern to conservation managers [63]. Moreover, we may well overlook important management insights if we assume that all populations are behaviourally homogenous. We have long recognized the importance of preserving genetic variation, and more recently conservation management practitioners have adopted the preservation of evolutionarily significant units, which recognizes the importance of local adaptation as well as the evolutionary processes that are responsible for generating this variation [64]. However, behavioural traditions are also important phenotypic variants that may (e.g. via gene–culture coevolution) or may not be aligned with underlying population genetic structure and are often of adaptive importance to the local population (see [65] for a review). Preserving the mechanisms that generate culture is clearly important, but there may be instances where the cultural trait itself might need to be preserved. If key local cultural traits are lost then they may never be recovered and if they are important to fitness, their loss may lead to local extirpation. Moreover, recognizing and conserving such behavioural variation for its own inherent value is worthwhile in its own right under a broader natural heritage conservation umbrella. For some species, this may be idealistic rather than realistic given limited conservation budgets.

Anthropogenic impacts such as climate change often result in shifts in the environment that outstrip the biological capacity for adaptation via natural selection. This is particularly the case in long-lived organisms with low rates of reproduction and long generation times. Unfortunately, the life-history characteristics of many larger, vulnerable fishes fall into this category, with longevity frequently exceeding several decades. Buffalofish (*Ictiobus cyprinellus*), lungfish (*Neoceratodus fosteri*) and sturgeon (*Huso huso*), for example, all have longevities that may exceed 100 years and many reef fish live for several decades. In these instances, social learning may provide an avenue for rapid adaptation to shifting environmental conditions at a pace far quicker than classical genetic adaptation, which takes multiple generations. This is because novel solutions to environmental threats can rapidly spread through a population via horizontal transmission and may result in the establishment of new cultural variants via vertical transmission. Thus, rapid learning via social learning might enhance behavioural flexibility, leading to greater population resilience to anthropogenic change. An alternative argument is that change might happen too fast for culture to track, so existing cultural traditions become maladaptive. This is likely to be very important when conditions at traditional feeding or breeding grounds change, for example. In this instance, there is a mismatch between contemporary environmental conditions and the behaviour maintained by cultural inheritance [16]. In this scenario, the existence of strong cultural traditions (cultural conservatism) may impede the capacity for adaptation, resulting in a cultural trap [9]. Knowing just how fish will react to rapid environmental change will likely require managers to take social learning and culture into account, since these processes can clearly facilitate or impede adaptation and population resilience.

It is apparent that understanding social learning and fish culture may be useful in the context of conservation management, but what about fisheries management and stock assessment? At the very least, many of the same arguments apply and thus it is also important to recognize local cultural traditions in this context. Stock assessment, for example, is typically done at large spatial scales, but by doing so fails to consider smaller-scale phenotypic variation which may have considerable management implications. Social learning and culture also have implications for demographic processes, population structure and niche separation [9,65,66], which are key factors in stock assessment methodologies. There are further considerations in the context of fisheries management, which often relies on setting quotas or size limits as management tools. Fishing practices often deliberately target larger, older fish that are the holders of cultural information. As such, fisheries that target older individuals may inadvertently be removing cultural/traditional knowledge from the population, which may ultimately lead to population collapse [67]. It is increasingly apparent that many commercially important fish stocks have complex migration patterns that may well be culturally inherited. Ferno *et al*. [68], for example, suggest that many fish species that are targeted by fisheries are long-lived and thus probably have significant cultural contributions to their behaviour. Herring (*Clupea harengus*) schools, while probably not lead by the mythical herring king, do seem to be influenced by inter-cohort learning whereby the young cohort learn migration routes by following older cohorts [69]. There is good evidence that when there is strong young cohort recruitment into the population, this coincides with shifts in the location of traditional overwintering grounds, probably because the leadership of the older cohorts is diluted [70]. Similarly, individual cod (*Gadus morhua*) repeatedly home to the same spawning ground, the location of which is learned by following older fish on a migration ‘highway’ [71]. Social learning is particularly important in these contexts because the probability of inexperienced individuals stumbling upon suitable spawning and feeding grounds by chance is very low owing to the large spatial scales involved. In both cases, there have been dramatic collapses of stocks that were not predicted by traditional fisheries models. However, such collapses are more likely if key information like the location of high-quality feeding and breeding grounds is transmitted culturally. This can occur, for example, because cultural conservatism or culture conformity can limit the capacity for a population to track environmental change or because the transmission chain becomes disrupted through a reduction in population size, disruption of social structure or the removal of cultural leaders [9,72,73]. Once lost, cultures may never reestablish, as has occurred in North Atlantic Right whales, *Eubalaena glacialis*, post-whaling [74]. This would also explain why certain fish stocks have not recovered as expected despite years of fishing moratoriums.

Lastly, we can use our knowledge of social learning for conservation/fisheries management in the context of rearing fishes in captivity for release in the wild to either bolster existing stocks or generate new ones (reintroductions or translocations) ([75–77]; see also Greggor [78], this issue). In many instances, restocking has been used as the primary conservation method to sustain threatened or endangered fish species (e.g. [79,80]). Each year, tens of billions of fish are reared in captive aquaculture facilities for release into the wild. In North America for example, about 5.5 billion Pacific salmon, *Oncorhynchus* spp, are released annually [81]. The trouble is that the post-release survival rate of hatchery-reared fishes is typically very low, rarely exceeding 5% [82]. Thus, stock enhancement rarely makes a significant contribution to population sustainability [83,84]. The main issue is that captive-reared fishes develop maladaptive traits that make them unsuitable for survival in the wild owing to the mismatch between captive rearing conditions and the real world. This is clearly an important management issue, but it also has tremendous animal welfare implications that are seldom considered in the context of hatchery releases. Thus, there is room for significant improvements on this rate by employing pre-release training, often referred to as life skills training [75]. Here, the objective is to generate wild-like behaviour, morphology and physiology in captive-reared fish. A recent meta-analysis suggests that these types of conditioning programs significantly improve the fitness of hatchery-reared fishes [85].

While it is theoretically possible to train every single fish prior to release, it is certainly not logistically feasible. It simply takes too long. Fortunately, this is not necessary since we can use social learning to ensure that the required behavioural skills or knowledge spreads rapidly through the captive population. The typical scenario requires the training of a small proportion of the fish to recognize predators or live prey, for example. These ‘demonstrator’ individuals are then seeded into naive groups of fish and then demonstrate the behaviour to the naive fish, which subsequently learn the behaviour. By way of example, Brown and Laland [31] paired naive hatchery-reared Atlantic salmon (*Salmo salar*) with demonstrators that had been pre-trained to recognize and eat live prey. After 6 days of watching demonstrators attacking live prey, all naive observers had learnt the novel foraging behaviour, whereas only about half of those fish that had no demonstrator had eaten any live prey during the same period. Later experiments showed that the naive fish could be trained to feed on the surface or the benthos [86], illustrating that refinements can be made to the training protocol to ensure that the novel behaviour is ecologically relevant. Rapid learning as a result of social learning protocols was also shown in Atlantic cod learning to associate food with an audible tone. It took a week to train naive individuals in the control condition (individual learning), but just 2 days when a demonstrator was present [87]. Similar sorts of experiments have been conducted to teach hatchery-reared salmonids to recognize predators (e.g. [79]). Patten [88] found that if naive and experienced fish were mixed then their survival rate was 71%, similar to experienced fish (75%), but when tested alone it dropped to 46%, suggesting the social transfer of anti-predator behaviour from experienced to naive fish. While the theory and lab experiments demonstrating the utility of social learning to train hatchery-reared fishes have been around for several decades, practitioners have been slow to apply it at scale. Nonetheless, there have been several success stories. Queensland Fisheries, for example, used social learning training protocols to improve the post-release survival of the iconic Murray cod (*Maccullochella peelii*) by fourfold over control fish [89]. The approach adopted by Hutchinson and colleagues [89] makes use of the smell of alarm cues released from injured conspecifics, which fish then associate with the smell of a predator [34]. The alarm cue acts as a social contagion that rapidly spreads through groups of naive fish.

One of the outstanding management issues is that we still know so little about the natural behaviour of fishes in the wild. This is true even for heavily exploited species. While there has been a great deal of research on the mechanisms of social learning in fishes from lab studies on sticklebacks and guppies, our understanding of how this translates to wild fish is still poor. There are some compelling studies on a few coral reef fish where the evidence for culture in migration is extremely convincing [53,54,57–59], but these studies generally follow the behaviour of fish over reasonably small spatial scales. In contrast, ocean-going fish such as commercial species in the North Atlantic move over tens of thousands of kilometres, making it extremely challenging to study them. Somewhat ironically, these fish have been the target of commercial fishing for hundreds of years (e.g. cod and herring) and their fisheries generate tens of millions of dollars annually, yet there are still huge gaps in our understanding of what drives their movement and migration. To some extent, modern technology may come to the rescue as our capacity to tag and track animals in the ocean improves. In the meantime, fisheries and conservation managers should assume that many of these species do rely on social learning and culture for survival and reproduction and manage them accordingly. Some classic examples come from salmonids and sturgeons, both of which are heavily exploited, with many of them threatened from over-fishing. In both cases, there is evidence of population-specific behaviour indicative of local adaptation and or culture, and there are suggestions that migration timing may well be triggered by social cues [90–92]. In these instances, given the holes in our knowledge for all species, one might rely on phylogenetic inference and suggest that social learning and culture are likely important to all species in both of these families.

## 5. Conclusion

The importance of recognizing social learning and culture in conservation management is growing rapidly [8,9,63,65] but it has received far less attention in fishes. Several decades of research demonstrate that fish are capable of complex social learning, upon which culture critically depends. Traditions of socially transmitted behaviours can be established under experimental conditions and most significantly, there is convincing evidence of cultural transmission of local migration routes in several reef fish species as well as many commercially important fishes in the Northern Hemisphere. Given how common local diel and seasonal migration behaviours are in fishes, it seems plausible that social learning of route pathways and of feeding and breeding ground locations may play a role in shaping the behaviour of a wider range of species than is currently recognized. Here, we suggest that knowledge of social learning and culture in fishes can not only guide conservation action but may also be important in a fisheries management context. There is a clear and present need for further research in this area that can be facilitated by modern tagging technology and clever experimental manipulation. Lastly, there is growing use of using social learning in translocations and hatchery releases (see [78], this issue). While the impetus thus far has been on improving survival for management purposes, there are significant animal welfare issues associated with these practices that are seldom acknowledged.

## Data Availability

This article has no additional data.
